# Prognosis of Cyclic Vomiting Syndrome

**DOI:** 10.15844/pedneurbriefs-30-1-5

**Published:** 2016-01

**Authors:** J. Gordon Millichap

**Affiliations:** 1 Division of Neurology, Ann & Robert H. Lurie Children's Hospital of Chicago, Chicago, IL; 2 Departments of Pediatrics and Neurology, Northwestern University Feinberg School of Medicine, Chicago, IL

**Keywords:** Vomiting, Migraine, Valproic Acid, Adrenocorticotrophic Hormone, Antidiuretic Hormone

## Abstract

Investigators from Teikyo University School of Medicine, Tokyo, Japan, evaluated the clinical features, prognosis, and prophylaxis of cyclic vomiting syndrome and the relationship between the syndrome and levels of adrenocorticotropic/antidiuretic hormones (ACTH/ADH).

Investigators from Teikyo University School of Medicine, Tokyo, Japan, evaluated the clinical features, prognosis, and prophylaxis of cyclic vomiting syndrome and the relationship between the syndrome and levels of adrenocorticotropic/antidiuretic hormones (ACTH/ADH). In 31 patients with the syndrome admitted between 1996 and 2008 the diagnosis was based on criteria of the 2nd edition of the International Headache Classification, and 25 were followed until 2013. Abdominal pain, diarrhea, and headache were associated in 23, 10, and 18 patients, respectively. A family history of migraine was identified in 13 (42%) patients. The median duration of the disorder was 66 (3-179) months, and 44% (n=11) developed migraine. Prophylactic therapy was started for 18 patients with severe symptoms; valproic acid (VA), VA with phenobarbital, phenobarbital, and amitriptyline were effective in 9, 4, 3, and 1 patient, respectively. The median frequencies of attacks were 0.75 and 0.33 per month with and without prophylaxis, respectively. Abnormally high levels of ACTH (n=17) and ADH (n=18) were found among the 25 patients with data available. Attack duration was correlated with levels of ACTH (p=0.0084) and ADH (p=0.0031). ADH levels in patients with bilious vomiting were higher than in those without (p=0.048). Most patients with cyclic vomiting syndrome recovered completely and responded to prophylactic therapy, but half of the patients developed migraine. [1]

COMMENTARY. The cause of cyclic vomiting syndrome is usually undetermined and is made by exclusion following extensive testing. In a study of 106 patients aged <21 years at the Cleveland Clinic, neuroimaging revealed intracranial abnormalities in <10% of patients, none of which explained the vomiting. Abdominal ultrasound revealed abnormalities in 15% of patients during an acute episode and 7% when well. An upper gastrointestinal series was normal in all of 61 patients tested. Laboratory testing in 92% of patients revealed abnormalities suggesting mitochondrial dysfunction in 38% [2]. A relationship between mitochondrial dysfunction and migraine is supported by biochemical, muscle biopsy, genetics, and therapy with riboflavin, coenzyme Q10, niacin, carnitine, and topiramate [3]. That cyclic vomiting may represent a form of epilepsy in children was proposed in a report of 33 patients, 7 (21%) having a history of generalized tonic-clonic or complex partial seizures in addition, and 25 (76%) with seizure discharges in the EEG, some focal and predominantly temporal in localization [4]. Most of the EEGs were not recorded at the time of the vomiting, and in retrospect, some of these cases may be classified as a form of migraine. A response to antiepileptic medication was compatible with a diagnosis of epilepsy or migraine [5]. In a 1970s study of recurrent headaches and EEG abnormalities in 100 children, phenytoin controlled migraine in 77% and the response was unrelated to the degree of EEG abnormality. In the present report of cyclic vomiting syndrome, the cause appears to favor a migraine, but the EEG and family history of epilepsy are not recorded, despite a positive response to treatment with the antiepileptic medication, valproate. Future search for the cause or causes of cyclic vomiting should consider the inclusion of the EEG.
